# The Cæsarean Operation in the U. S.

**Published:** 1872-04

**Authors:** Robert P. Harris


*The Cæsarean Operation in the United States.* By ROBERT P. HARRIS, M.D.

This is the title of an historical monograph of some thirty pages in length, which has just appeared in the *Am. Journal of Obstetrics*, as a portion of the proceedings of the Philadelphia Obstetrical Society; to be followed in the February number of the said journal by an abstract of cases, published and unpublished, which have been, many of them, rescued from obscurity after a tedious research in public and private libraries, and by an extensive correspondence embracing every State in the Union. The subject of the paper is one of considerable interest, both to the surgeon and the obstetrician, and was undertaken by Dr. Harris for two reasons: 1st. To show the success of the Cæsarean operation in our country; and 2nd, the importance of resorting to the use of the knife early, if it is to be employed with a reasonable hope of success.

Up to the present writing, we are informed by Dr. Harris, that he has collected sixty cases of the operation; which resulted in saving the lives of thirty-two women and twenty-seven children; the last operation dating November 23, 1871. Of the sixty operations, only seventeen were performed with a reasonable degree of promptness, calculated either by hours, or based upon the condition of the patient, the latest being twenty-four hours after the commencement of labor. These resulted in the saving of twelve women, 70 1-17 per cent., and fourteen children, 82 6-17 per cent. All the children were alive when delivered save one, which was born of a sickly woman, having a fibrous tumor and affected with intermittent fever. Two died soon after delivery, one being deformed in the lower extremities, and both very feeble. Of the five women who died, two fell victims to peritonitis (one having been operated upon twice before); one to obstruction of the bowels; one to secondary shock; and one to "irritative fever," at the end of three weeks.

These results establish very conclusively the value of operating early, and before the woman has been injured by craniotomy or exhausted by other fruitless efforts at delivery. In contrast with this degree of success it may be well to state that there were eightyeight Cæsarean operations performed in Great Britain and Ireland from January, 1738, to March, 1866, before twelve women were saved.

Dr. Harris has collected one hundred and six cases for Great Britain and Ireland, showing a saving of sixty children and but eighteen women. From 1822 to 1852 inclusive, twenty-six operations are recorded as performed in the United States, resulting in the saving of eighteen women and nine children. Ten of the twenty-six women were operated upon on the first day of labor, resulting in saving eight, with seven of the children. English statistics point to a more favorable result than American as regards the children, but to a much less favorable result as regards the women. This is ascribable in part to delay in most cases, but is no doubt also in great measure owing to the large proportion of subjects whose pelvic deformity arises from the existence of malacosteon, not a single case of which is recorded in the table for the United States, the deformities (33) being all due to rachitis in childhood. It is also probable that a cold, damp climate is unfavorable to success. One-third of our operations have been performed in the States of Louisiana (8), Alabama (7), and Mississippi (5), resulting in saving twelve, or sixty per cent. of the women, and ten children. One woman, in Mississippi, was operated upon three times, and died of peritonitis on the third occasion, although there was no delay in making the section. The first and second operations took place in warm weather, and cold lotions were applied to the abdomen; the last was in October,—a much less favorable season of the year in that locality.

From the investigations of Sir Charles Bell, and more recently of Spencer Wells, and others, into the condition of the uterine wound after the completion of the operation, it has become a question of some moment to decide whether or not it is advisable to close this incision by sutures; and this matter has assumed a definite experimental character on both sides of the Atlantic during the last four years. Of nine examples collected from British tables, eight belong to this period, and of seven, in Dr. Harris's table, six are in the same category. Two cases in each list had a favorable termination, silver sutures being employed in two; linen thread in one; and the long-tailed removable uninterrupted silk suture in the other. Two American women, recently reported in good health, who it is not at all probable could have been saved but for this expedient, have carried, without any perceptible inconvenience, the one six silver sutures, and the other five thread ones, for the last four years.

The results of ovariotomy have demonstrated that traumatic peritonitis is not so much to be dreaded as the same inflammation from other causes; and those of the Cæsarean operation have shown that hæmorrhage, exhaustion, and the escape of the lochia into the peritoneal cavity, are more to be feared than the injury to the peritoneum by the knife, especially when the last is not favored by exhaustive delay in resorting to it.

The value and safety of the uterine suture have yet to be shown, as it is impossible from the few cases in which it has been employed to say what may be anticipated from its use. It is certain that some cases might be saved by it that could not be without it, but it is equally certain that its influence as a cause of peritonitis has not been as yet estimated; it is probable that this may be more than balanced by the salutary effects of checking hæmorrhage and preventing the entrance of uterine discharges into the abdominal cavity.

What would be the effect of pregnancy upon imbedded sutures in the uterine walls has still to be shown, as we are not aware that any such test has yet been made. To obviate the possibility of risk in this event, various forms of removable suture have been proposed, but the difficulty of using them, as compared with the interrupted suture cut close, has prevented a full trial of their merits.—*Med. Times*, Jan. 2, 1872.

